# Local and regional temporal trends (2013–2019) of canine *Ehrlichia* spp. seroprevalence in the USA

**DOI:** 10.1186/s13071-020-04022-4

**Published:** 2020-03-30

**Authors:** Jenna R. Gettings, Stella C. W. Self, Christopher S. McMahan, D. Andrew Brown, Shila K. Nordone, Michael J. Yabsley

**Affiliations:** 1 Southeastern Cooperative Wildlife Disease Study, University of Georgia, Athens, 30602 USA; 2 Arnold School of Public Health, University of South Carolina, Columbia, 29208 USA; 3 School of Mathematical and Statistical Sciences, Clemson University, Clemson, 29634 USA; 4 Comparative Medicine Institute, North Carolina State University College of Veterinary Medicine, Raleigh, 27607 USA; 5 Warnell School of Forestry and Natural Resources, University of Georgia, Athens, 30602 USA

**Keywords:** *Ehrlichia canis*, *Ehrlichia chaffeensis*, *Ehrlichia ewingii*, Temporal trends, USA, Vector-borne

## Abstract

**Background:**

In the USA, there are several *Ehrlichia* spp. of concern including *Ehrlichia canis*, *Ehrlichia ewingii*, *Ehrlichia chaffeensis*, *Ehrlichia muris eauclarensis*, and “Panola Mountain *Ehrlichia*”. Of these, *E. canis* is considered the most clinically relevant for domestic dogs, with infection capable of causing acute, subclinical, and chronic stages of disease. Changes in climate, land use, habitats, and wildlife reservoir populations, and increasing contact between both human and dog populations with natural areas have resulted in the increased risk of vector-borne disease throughout the world.

**Methods:**

A Bayesian spatio-temporal binomial regression model was applied to serological test results collected from veterinarians throughout the contiguous USA between January 2013 and November 2019. The model was used to quantify both regional and local temporal trends of canine *Ehrlichia* spp. seroprevalence and identify areas that experienced significant increases in seroprevalence.

**Results:**

Regionally, increasing seroprevalence occurred within several states throughout the central and southeastern states, including Missouri, Arkansas, Mississippi, Alabama, Virginia, North Carolina, Georgia and Texas. The underlying local trends revealed increasing seroprevalence at a finer scale. Clusters of locally increasing seroprevalence were seen from the western Appalachian region into the southern Midwest, along the Atlantic coast in New England, parts of Florida, Illinois, Wisconsin and Minnesota, and in a couple areas of the Mountain region. Clusters of locally decreasing seroprevalence were seen throughout the USA including New York and the mid-Atlantic states, Texas, the Midwest, and California.

**Conclusions:**

Canine *Ehrlichia* spp. seroprevalence is increasing in both endemic and non-endemic areas of the USA. The findings from this study indicate that dogs across a wide area of the USA are at risk of exposure and these results should provide veterinarians and pet owners with the information they need to make informed decisions about prevention of tick exposure.
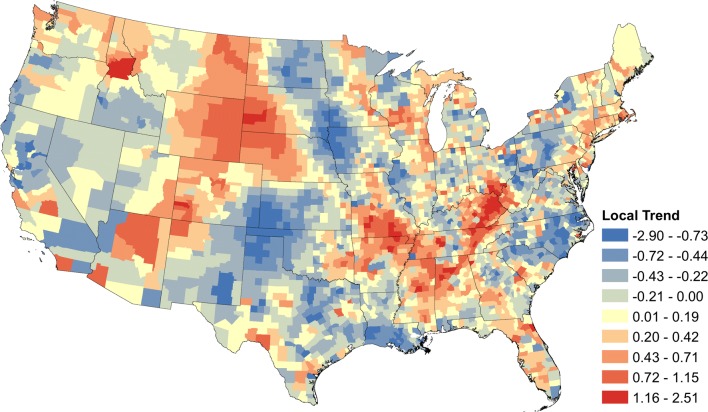

## Background

The predominately recognized agents for canine ehrlichiosis in the USA include *Ehrlichia canis*, *Ehrlichia ewingii* and *Ehrlichia chaffeensis* [1]. Despite their shared genus, they are transmitted by different vectors: *Amblyomma americanum* is the primary vector of *E. ewingii* [2] and *E. chaffeensis* [3], and *Rhipicephalus sanguineus* is the primary vector of *E. canis* [4]. The distribution of infected dogs follows that of the associated tick vectors, and the seroprevalence of antibodies against *E. ewingii* or *E. chaffeensis* generally exceeds that of *E. canis* [5, 6]. Recent evidence suggests that dogs are also susceptible to infection by *Ehrlichia muris*, believed to be transmitted by *Ixodes scapularis* [7] and “Panola Mountain *Ehrlichia*”, which is transmitted by *A. americanum* [8]. In 2019, over 200,000 dogs tested positive for antibodies against *Ehrlichia* spp. within the USA out of 7,056,709 tested [9]. In Canada, over 1000 dogs tested positive out of 168,216 tested, but this is likely an underestimate as these data have just recently started being collected [9].

Several of these *Ehrlichia* spp. also infect people and the incidence of human ehrlichiosis, similar to other vector-borne diseases, has been reported to be increasing over the past several years [10]. In humans, *E. chaffeensis* is the most commonly reported infection followed by *E. ewingii*, but sporadic infections with *E. muris eauclarensis* and “Panola Mountain *Ehrlichia*” have also been reported [11, 12]. Changes in the number of Centers for Disease Control and Prevention (CDC)-reported confirmed and probable human ehrlichiosis cases [13] may be associated with the northward expansion of *A. americanum* and *E. chaffeensis* from their historical range in the southern USA [14], with newly established counties reported as far north as South Dakota and New Hampshire [15]. Trends for the other ehrlichiosis agents in humans and dogs are not known. *Rhipicephalus sanguineus* is found worldwide [16], but different lineages, temperate and tropical, have different geographical distributions [17, 18]. The tropical lineage is associated with outbreaks of Rocky Mountain spotted fever in Mexico [19], but until recently has not been known to be present in the USA. In 2018, tropical-lineage ticks were deemed to be established along the entire border from San Diego, California to western Arizona, and as far north as Los Angeles, California [18]. Whether this will impact the risk of exposure to *E. canis* in the USA remains to be seen.

Veterinarians often test for the presence of *Ehrlichia* spp. antibodies during canine annual wellness exams, resulting in a robust database of seroprevalence data that enables longitudinal analysis. The availability of over 30,000,000 test results, aggregated at a county and monthly level for vector-borne pathogen exposure in dogs since 2013, allowed us to evaluate temporal trends in the seroprevalence of *Ehrlichia* spp. and determine where risk of exposure is increasing or decreasing across the USA. This analysis is intended to enable veterinarians and pet owners to determine which preventative practices are best for their patients and pets.

## Methods

### Data

A total of 31,200,847 tests were reported across the contiguous USA from January 2013 to November 2019 by IDEXX Laboratories, available from [9]. There were 908,619 positive tests, yielding an overall raw seroprevalence of 2.91%. Results are from the SNAP® 4Dx® Plus test (IDEXX Laboratories, Inc. Westbrook, ME) which detects antibodies against *Ehrlichia* spp. as well as antibodies against *Anaplasma* spp. and *Borrelia burgdorferi* and antigen from *Dirofilaria immitis*. No other testing modalities are included in these data. Released in early 2012, the SNAP® 4Dx® Plus test includes three antigens for the detection of exposure to *Ehrlichia* spp.: p28 (*E. ewingii*) and p30/p30-1 (*E. canis*) [20]. There is also evidence of cross-reactivity with *E. chaffeensis* [21]. This test is used commonly by veterinarians throughout the USA both for annual screening during wellness examinations and for diagnosis of suspected vector-borne illness. Results from both veterinary clinics and IDEXX reference laboratories were collated automatically into a centralized database, from which aggregate data were provided to the investigators at a county and monthly scale. Importantly, the reported county is that of the clinic, and no patient histories are known.

**Fig. 1:**
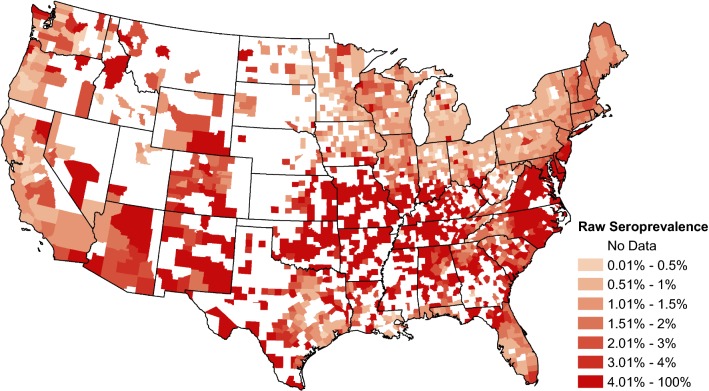
The raw canine *Ehrlichia* spp. seroprevalence from January 2013 to November 2019. Seroprevalence is calculated as the sum of positive tests for the study period divided by the sum of all tests. White counties are those which did not report any tests

Figure 1 displays the number of seropositive tests from each county divided by the number of tests performed in that county; data are aggregated over the study period. Counties which did not report any tests are shown in white.

### Model

We used the Bayesian spatio-temporal binomial regression model developed in [22] to model these data. Let $y_{st}$, $n_{st}$, and $p_{st}$ denote the number of seropositive tests, total number of tests, and seroprevalence (respectively) from county $s$ in month $t$. Assume that

1 $$\begin{array}{*{20}c} {y_{st} |n_{st} ,p_{st} \sim Binomial\left( {n_{st} ,p_{st} } \right) } \\ \end{array}$$

that is, conditional on $n_{st}$ and $p_{st}$, $y_{st}$ follows a binomial distribution with $n_{st}$ trials and a success probability of $p_{st}$. We modeled the seroprevalence *via*

2 $$\begin{array}{*{20}c} {g\left( {p_{st} } \right) = \eta_{st} = \delta + \beta_{s} t + \xi_{st} ,} \\ \end{array}$$

where $g^{ - 1} \left( \cdot \right)$ is the logistic link function, $\delta$ is a global intercept parameter, $\beta_{s}$ is the regression coefficient for county $s$, and $\xi_{st}$ is a spatio-temporal random effect for county $s$ in month $t$.

We interpret $\beta_{s}$ as the regional trend at county $s$ (note that the $\beta_{s} '$s vary by county). Positive values of $\beta_{s}$ indicate an increasing regional trend at county $s$, while negative values indicate a decreasing trend. A Gaussian predictive process (GPP) [23] was used to model the $\beta_{s}$’s, allowing them to change smoothly over the study area. GPPs borrow information over space, so that the regional trend at county $s$ is influenced by trends from a relatively large surrounding. GPPs are determined by a mean function, a covariance function, and a set of spatial knot locations. The specifications used here are identical to those used in [22].

The data present with heavy spatio-temporal dependence. Neglecting to account for this dependence can lead to biased estimation and unreliable inference. The spatio-temporal random effects are included to model this dependence *via* a vector autoregression with an embedded conditional autoregressive (CAR) structure similar to that found in [24] and [25]. For more on CAR models see [26]. For each month $t$, define the vector ${{\mathbf{\phi_t}}} = \left( {\phi_{1t} ,\phi_{2t} , \ldots ,\phi_{St} } \right)^{\prime }$, where $S$ is the total number of counties. We assume that the $\mathbf{\phi_t}$’s are independent realizations from a CAR model, that is, $\phi_{t}$ follows a multivariate normal distribution with mean $0$ and variance-covariance matrix given by $\tau^{2} ({\mathbf{D}} - \rho {\mathbf{W}})^{ - 1}$. Here $\tau^{2} > 0$ is a marginal variance parameter, $\rho \in \left( {0,1} \right)$ influences the degree of spatial correlation, ${\mathbf{W}}$ is the county adjacency matrix (i.e. the $\left( {i,j} \right)$th entry of ${\mathbf{W}}$ is 1 if counties $i$ and $j$ share a border and 0 otherwise), and ${\mathbf{D}}$ is a diagonal matrix whose $i$th diagonal entry is equal to the number of counties sharing a border with county $i$. Define ${\varvec{\upxi}}_{t} = \left( {\xi_{1t} ,\xi_{2t} , \ldots ,\xi_{St} } \right)'$ to be the vector of spatio-temporal random effects from month $t$. Our assumed vector autoregression is given by ${\varvec{\upxi}}_{1} = \mathbf{\phi}_{1}$ and ${\varvec{\upxi}}_{{\mathbf{t}}} = \zeta {\varvec{\upxi}}_{{{\mathbf{t}} - 1}} + {{\mathbf{\phi_t}}}$ for $t \ge 2$. Here $\zeta \in \left( { - 1,1} \right)$ is a temporal correlation parameter.

As the model is Bayesian, fully specifying the model necessitates assigning prior distributions to all unknown parameters. The priors used here are identical to those used in [22]. Markov chain Monte Carlo (MCMC) methods are used to sample the model parameters from the posterior distribution, and estimation and inference are performed using this sample in the typical manner. In particular, the posterior mean estimate of the regional trends, (the $\beta_{s}$ terms) is shown below.

We used the posterior parameter sample to estimate the local trends as follows. For each MCMC parameter sample $g$, and each county $s$, fit the following ordinary least squares model

3 $$\eta_{st}^{(g)} = \alpha_{0s}^{(g)} + \alpha_{1s}^{(g)}t + \epsilon_{st}^{(g)}, \quad t = 1, \ldots ,T.$$

Here $\eta_{st}^{\left( g \right)}$ is the value of $\eta_{st}$ calculated using the values of $\delta ,\beta_{s}$, and $\xi_{st}$ obtained on the $g$th iteration of the posterior sampling algorithm after convergence, the ${{\epsilon }_{st}}$ are independent and identically distributed normal random variables, and $T$ is the total number of months in the study period. For each county $s$, the set of $\widehat{\alpha }_{1s}^{\left( g \right)}$’s is a posterior sample of the local trend parameter at county $s$. Estimation and inference for the local trends is conducted using this sample in the usual way. For more information on this model, as well as more details on the calculations of the regional and local trends, see [22].

### Assessing model accuracy

In order to assess the predictive performance of our model, we provide a receiver operating characteristic (ROC) curve. ROC curves plot the sensitivity (the percent of positive tests correctly predicted as positive by the model) against 1 minus the specificity (the percent of negative tests correctly predicted as negative by the model). See [27, 28] for more on ROC curves. The area under the ROC curve, or the AUC, is commonly used to assess the predictive performance of models for binary data, with an AUC value greater than 0.7 generally considered indicative of an acceptable model [29].

## Results

### Model accuracy

**Fig. 2:**
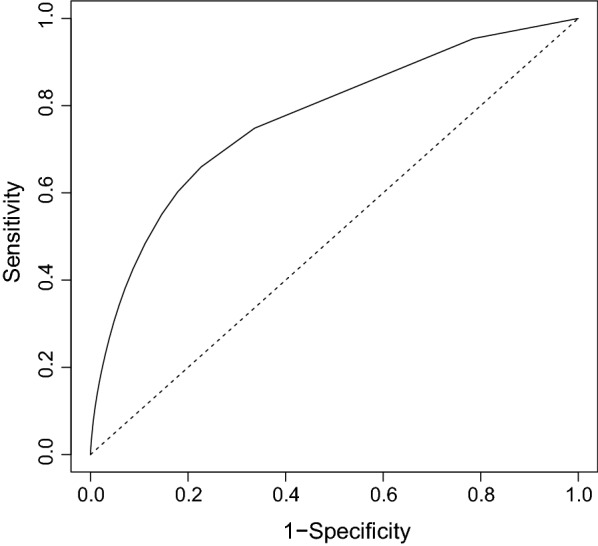
Receiver operating characteristic (ROC) curve. Plot of the sensitivity (the percent of positive tests correctly predicted as positive by the model) against one minus the specificity (the percent of negative tests correctly predicted as negative by the model)

The ROC curve is shown in Fig. 2. Our model has an AUC of 0.77, indicating that is has good predictive performance.

### Regional temporal trends

**Fig. 3:**
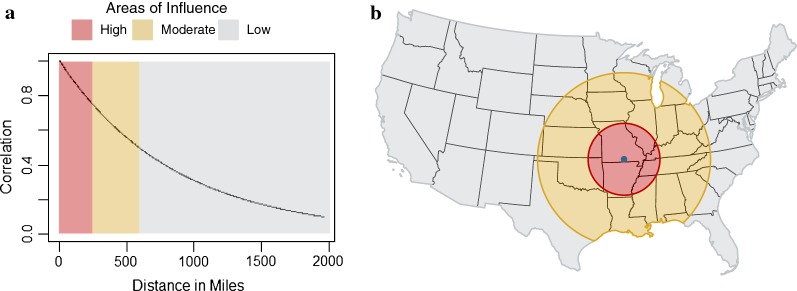
**a** The correlation between the trend parameter for canine *Ehrlichia* spp. for a given county and any other county is determined as a function of distance between those two counties. The curve represents the decaying influence as distance increases between the two counties. This is depicted in panel **b** using Howell County, MO as an example. Counties within the red circle have much greater influence over the regional trend estimate for Howell County than those within the grey area

This analysis provides two different estimates of the change in *Ehrlichia* spp. seroprevalence in dogs over the study period. The regional trends provide a large-scale estimate of the trend without having to report a fixed national estimate or selecting arbitrary administrative borders for aggregation. Instead, the regional trend estimate for each county was estimated by aggregating data from surrounding counties and allowing near counties to influence the estimate more than those counties farther away. This continuously diminishing influence with increasing distance is formally represented in Fig. 3. For demonstrative purposes only, three areas of high, moderate, and low influence were chosen to represent the distance at which the correlation is above 0.75 (high), between 0.75 and 0.5 (moderate), and below 0.5 (low). The corresponding distances are 0–246 miles (0–396 km), 247–592 miles (397–953 km), and > 592 miles (> 953 km), respectively. The example is extended to the map (Fig. 3b) using Howell County, Missouri as the county of interest.

**Fig. 4:**
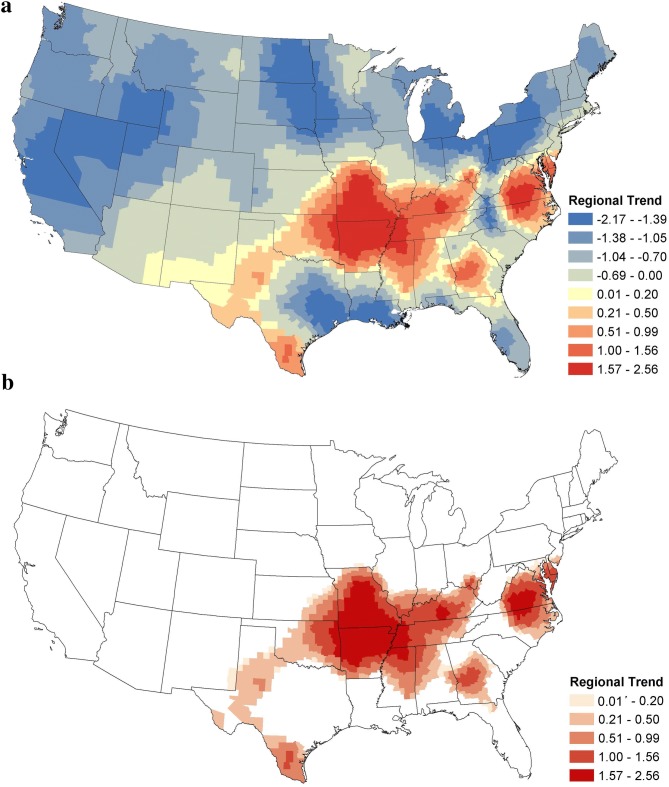
Posterior mean values of the regional temporal trend parameter for canine *Ehrlichia* spp. seroprevalence. **a** Posterior means of the regional temporal trend parameter, $\beta_{s}$ from Equation 2 for all counties. **b** Counties in red are those for which the 95% credible interval was strictly positive

Regional trends for canine *Ehrlichia* spp. seroprevalence between January 2013 and November 2019 are shown in Fig. 4a. Displayed are the estimated posterior mean values of the regional temporal trend parameter $\beta_{s}$ from Equation 2. Positive values in red tones indicate regions of increased seroprevalence, while the negative blue tones indicate decreased seroprevalence. Those with a statistically significant increase are shown in Fig. 4b. Significance was assessed with 95% credible intervals. Any county for which the credible interval of the temporal trend parameter was strictly greater than 0 was deemed to be a region of significant increase.

As shown in Fig. 4, four regional coalescing foci of positive regional trends were present: the Ozarks (Missouri, Arkansas and surrounding states), Virginia and North Carolina, Georgia and southern Texas. The Ozarks region experienced the largest positive trends, followed by Virginia and North Carolina, indicating the two areas of greatest increase. Cross-reactivity between *Ehrlichia* spp. precludes us from determining which species or vectors are driving these changes. No significant increase in *Ehrlichia* spp. seroprevalence was observed in the northern or western USA.

### Local temporal trends

**Fig. 5:**
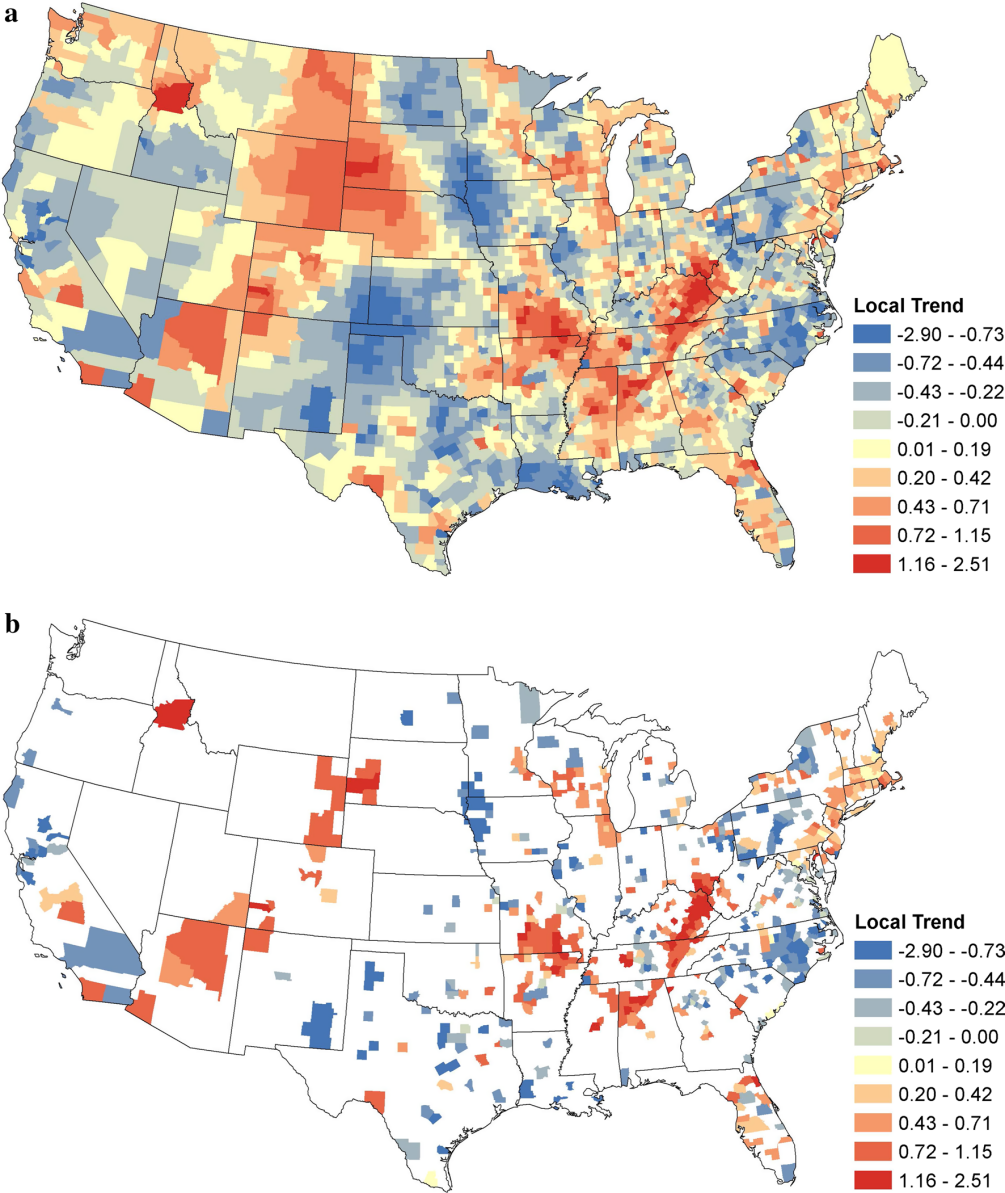
Posterior mean values of the local temporal trend parameter for canine *Ehrlichia* spp. seroprevalence. **a** Posterior means of the local temporal trend parameter for all counties. **b** Posterior means of the local temporal trend parameter $\alpha_{1s}^{{}}$ for counties in which the 95% credible interval did not contain zero

In addition to the regional trends, we obtained county-level trends by extracting $\widehat{\alpha }_{1s}^{\left( g \right)}$ from Equation 3. The result is shown in Fig. 5a, with statistically significant positive and negative trends as determined by 95% credible intervals shown in Fig. 5b. The largest clusters of positive trends were present in the western Appalachian region and Missouri and Arkansas. Slightly positive trends extended along the Atlantic coast of the New England states. Other clusters were observed in Illinois, Wisconsin and Minnesota; Florida, and in a few areas of the Mountain states (Fig. 5b). Clusters of negative trends were evident throughout the mid-Atlantic states and New York, particularly North Carolina, Midwest, Texas and California. Counties with both positive and negative trends are scattered throughout the USA, demonstrating the heterogeneity of underlying temporal trends.

## Discussion

The two maps of canine *Ehrlichia* spp. seroprevalence temporal trends (Figs. 4 and 5) provide (i) a regional perspective that represents the changes that occurred in the population of dogs spanning multiple states, showing the large scale changes in seroprevalence; and (ii) a local perspective that represents changes that occurred within a county-level population of dogs. This local trend provides a more accurate estimation for the change in risk of exposure to the local population of dogs, though neither map can ascribe risk to an individual dog. Because we performed inference on the trends in over 3000 counties, we were faced with the well-known ‘multiple testing problem’, i.e. the inflation of group type I error rate to levels well above that of each individual test. However, this problem is mitigated through the use of a Bayesian hierarchical model, which has inherent protection against the multiple testing problem [30, 31]. We also show the significance of trends at the 1% significance level and the standard deviation of the trends in each county to illustrate a more conservative level of significance (Additional file 1: Figures S1, S2).

The interpretation of temporal trends for *Ehrlichia* spp. seroprevalence in dogs is complicated by the number of cross-reactive species within the genus. The test is labeled to detect *E. canis* and *E. ewingii* [32] but has evidence of frequent cross-reactivity with *E. chaffeensis* [21]. Cross-reactivity with other *Ehrlichia* spp., known or not recognized yet, is also important to consider. Prior studies reported the anomalous presence of *Ehrlichia* spp. seropositivity in dogs from the upper Midwest [33] that is now believed to be associated with the emergence of the recently recognized *E. muris eauclarensis* [7]. In addition, “Panola Mountain *Ehrlichia*” has been detected in dogs [34]. Only the case with *E.* *muris* reported a weak positive on the SNAP® 4Dx® Plus test. Additionally, while these pathogens are transmitted by different tick vectors, the vector ranges have extensive spatial overlap. *Ambylomma americanum* (vector of *E. ewingii* and *E. chaffeensis*) is predominately found in the southern states and along the Atlantic coast into Maine; while *R. sanguineus* (vector of *E. canis*) and is distributed worldwide [16] with higher numbers occurring in warmer climates [35]. Questing *Dermacentor variabilis* ticks in the USA have been found to be infected with *E. chaffeensis* and *E. ewingii* [36], although prevalence of infection may vary geographically as similar studies in Tennessee and Virginia did not detect *Ehrlichia* spp. in *Dermacentor* spp. ticks [37, 38]. The implications of these findings are unknown as only *E. canis* has been successfully transmitted by *D. variabilis* in an experimental setting [39]. *Dermacentor variabilis* is widely distributed throughout the USA, but there is no current evidence of range expansion. Its influence on the temporal trends of canine *Ehrlichia* spp. seroprevalence is unknown.

Prior seroprevalence studies conducted at the species level for canine ehrlichiosis help us to better understand the temporal trends. Among dogs with suspected tick-borne illness in the USA, Qurollo et al. [6] found that *E. ewingii* was most prevalent with 3.8% seropositive, followed by *E. chaffeensis* (3.1%) and then *E. canis* (1.8%). States with a higher *E. canis* seroprevalence as compared to either *E. ewingii* or *E. chaffeensis* include Alabama, Texas, Colorado and Minnesota (although Minnesota had a small sample size and only one dog tested positive). Another study performed with samples collected for multiple reasons (not necessarily from suspected tick-borne disease cases), also found that the seroprevalence of *E. canis* was higher in Texas compared to the other two pathogens [5]. This might suggest that the regional and local trends we observed in Texas, and possibly Colorado and Alabama, may be driven more by *E. canis*. In contrast, in both previous studies, the seroprevalence of *E. ewingii* and *E. chaffeensis* was higher compared to *E. canis* in Arkansas and Missouri and many of the surrounding states and in the Atlantic states. Overall, the seroprevalence of *E. ewingii* or *E. chaffeensis* was 3–30% higher than *E. canis* within the same state [5, 38]. These studies highlight the importance of longitudinal species-specific data to accurately understand the species-specific temporal trends, but data from these two studies do provide insight on which pathogens are likely to influence *Ehrlichia* spp. trends within a given region.

Although we detected increasing seroprevalence in several regions of the USA, the reasons for these increases cannot be inferred from this study alone and are likely multifactorial and region-specific. However, these results can help researchers build hypotheses and focus future studies in areas experiencing significant changes in order to determine factors associated with temporal trends. Increased prevalence could be associated with higher tick densities, increased prevalence of infected ticks, more frequent interaction with tick habitats, increased wildlife reservoirs or other changes in wildlife population diversity, decreased use of preventative measures, or changes in testing practices among veterinarians. For the pathogens transmitted by *A. americanum*, there is evidence of range expansion for the tick [15] which is believed to be related to, in part, reforestation and subsequent re-establishment of and increases in the white-tailed deer population [40]. The impact of climate change on habitat suitability is expected to allow further northward expansion of *A. americanum* [41]. Furthermore, risk of exposure is impacted by changes in the interface between developed and undeveloped landscapes, such as occurs in suburban areas. Populations of *A. americanum* can also be found in planned green spaces within the urban environment [42] and in residential suburban areas [43]. As dogs move with their owners in and out of these areas, their risk of exposure to ticks may change.

Regarding *E. canis*, there is also evidence of changes in *R. sanguineus* populations. As noted earlier, there are two lineages of this tick species, and evidence of northward range expansion of the tropical lineage has been found in parts of the southern USA, including California [18]. Detection of the tropical lineages have also been reported in Texas, Florida, Illinois and Arizona [44]. Experimentally, ticks from the tropical lineage have been shown to be a more competent vector for *E. canis*, compared to ticks from the temperate lineage [45]. In support of this finding are several studies that detected *E. canis* in the tropical lineages only [46, 47]. This information is particularly interesting in light of the evidence we present for the increasing trends of *E. canis* prevalence in those states in which the tropical lineage has been detected (Fig. 4).

Factors unrelated to the movement of pathogens and vectors should be considered with interpreting these results. Specifically, the impact of testing practices and preventative practices that reduce the risk of exposure to ticks. Throughout the study period, the number of tests performed within the USA has increased each year across all states [9]. This increase is believed to be associated with the willingness of veterinarians and pet owners to screen dogs for exposure to various vector-borne pathogens during annual wellness visits. As a result, areas with negative trends may be identified because a larger population of healthy and seronegative dogs are being tested over time, resulting in a “dilution” of the seroprevalence. Concurrently, the increase in wellness screening may have been accompanied by an increase in preventative measures, such as the use of acaricides, reducing the risk of exposure, and thus seroprevalence. Alternatively, in areas that do not routinely screen dogs, increased awareness of infection may result in increased testing of ill dogs, thus increasing the seroprevalence. This could reflect a true increase in seroprevalence but could also be an increase in detection of an infection that was already present. While we cannot control for testing and preventative use changes, continuing to monitor temporal changes and interpreting the trends in the context of other known information (e.g. vector range and species-specific distribution) will assist in the identification of regions experiencing real and clinically relevant changes in seroprevalence.

The analysis is limited in part by the population of dogs being tested. It is presumed that the majority are under the care of a veterinarian and may be more likely to receive some preventative care, such as acaricides to reduce the risk of tick bites or transmission of pathogens. Thus, the presented results may not represent dogs at higher risk of exposure within the USA (e.g. shelter dogs). Both the regional and local trends should be interpreted within the context of past and current prevalence [9]. The values of the trends in Figs. 4 and 5 are relative to the underlying prevalence. To elaborate, even small changes in areas of very low prevalence may have large trend values, while that same change in high prevalence areas may have a small trend value. Another limitation is the lack of knowledge about individual dogs in these data. No histories are known for travel, place of exposure, or reason for testing. Also, some dogs may be tested more than once. However, repeat testing and travel are believed to be a small portion of these data and the focus of the analysis is the temporal trends, which are going to be minimally influenced by these factors. Current practices among many animal shelters and rescues involve the translocation of dogs between different regions of the country. The predominate direction of these movements is from the southern states to the northern states, particularly the Northeast, but animals are also moved from Texas and California to midwestern or western states [48]. The impact of these movements on the prevalence of canine tick-borne disease is unknown at this time, but animals that are exposed in *Ehrlichia* spp. endemic areas may subsequently test positive in non-endemic areas. Owners and veterinarians should consider this when testing dogs that have been adopted from a shelter or rescue.

Several *Ehrlichia* spp. are zoonotic, and domestic dogs have been suggested to be effective sentinels for tick-borne pathogens including *Borrelia burgdorferi* (causative agent of Lyme disease) [49, 50] and *Rickettsia rickettsii* [51]. However, when interpreting our results, one should keep a few details in mind. Based on data discussed above, we believe that in the Midwest and other parts of the eastern/southeastern USA, much of the *Ehrlichia* spp. seroreactivity in dogs is due to exposure to *E. chaffeensis* and *E. ewingii* [5, 6]. Most cases of human ehrlichiosis are caused by *E. chaffeensis* [13], but cases of *E. ewingii* are reported annually in addition to the recently discovered *E. muris eauclarensis* [12]. The geographical distribution of reported human ehrlichiosis cases [13] closely matches that of canine *Ehrlichia* spp. seroprevalence [9]. This suggests that dogs in these areas may serve as sentinels for zoonotic ehrlichiae. However, in some regions (e.g. Arizona, Texas), reactivity is likely due to *E. canis* which is not considered a zoonotic pathogen. Future studies are needed to determine how well canine *Ehrlichia* spp. seroprevalence can estimate the risk of human ehrlichiosis cases and if there are similar spatio-temporal trends for both of these hosts.

## Conclusions

The study presented here highlights the regions of greatest concern for changing canine ehrlichiosis risks in the USA. Given the widespread distribution of the multiple vectors and species of *Ehrlichia*, nearly all dogs are at risk of exposure, but risk of infection is greatest in the Southeast and Midwest, particularly Missouri and Arkansas, and these regions are also the areas in which we report the largest increasing trends. Pet owners and veterinarians should always practice appropriate care in preventing exposure to ticks (e.g. tick preventatives, thorough examination for ticks, and avoiding tick habitat if possible), especially in areas with high risk.

## Supplementary information

**Additional file 1: Figure S1. a** Posterior mean values of the regional temporal trend parameter for canine *Ehrlichia* spp. seroprevalence for counties in which the 99% credible interval was strictly positive. **b** The posterior standard deviation of the regional temporal trend parameter, $\beta_{s}$ from Equation 2 for all counties. **Figure S2. a** Posterior mean values of the local temporal trend parameter $\alpha_{1s}^{{}}$ for canine *Ehrlichia* spp. seroprevalence for counties in which the 99% credible interval did not contain zero. **b** Posterior standard deviation of the local temporal trend parameter for all counties.

## Data Availability

The dataset analyzed during the present study are available from http://www.capcvet.org.

## Abbreviations

AUC: area under the curve
CAR: conditional autoregressive
CDC: Centers for Disease Control and Prevention
GPP: Gaussian predictive process
MCMC: Markov chain Monte Carlo
ROC: receiver operating characteristic
