# Supplementary figures and images for: Local and regional temporal trends (2013–2019) of canine Ehrlichia spp. seroprevalence in the USA
Source: Parasit Vectors. 2020 Mar 30;13:153. doi: 10.1186/s13071-020-04022-4 (PMC7106614; doi:10.1186/s13071-020-04022-4)

Figure S1

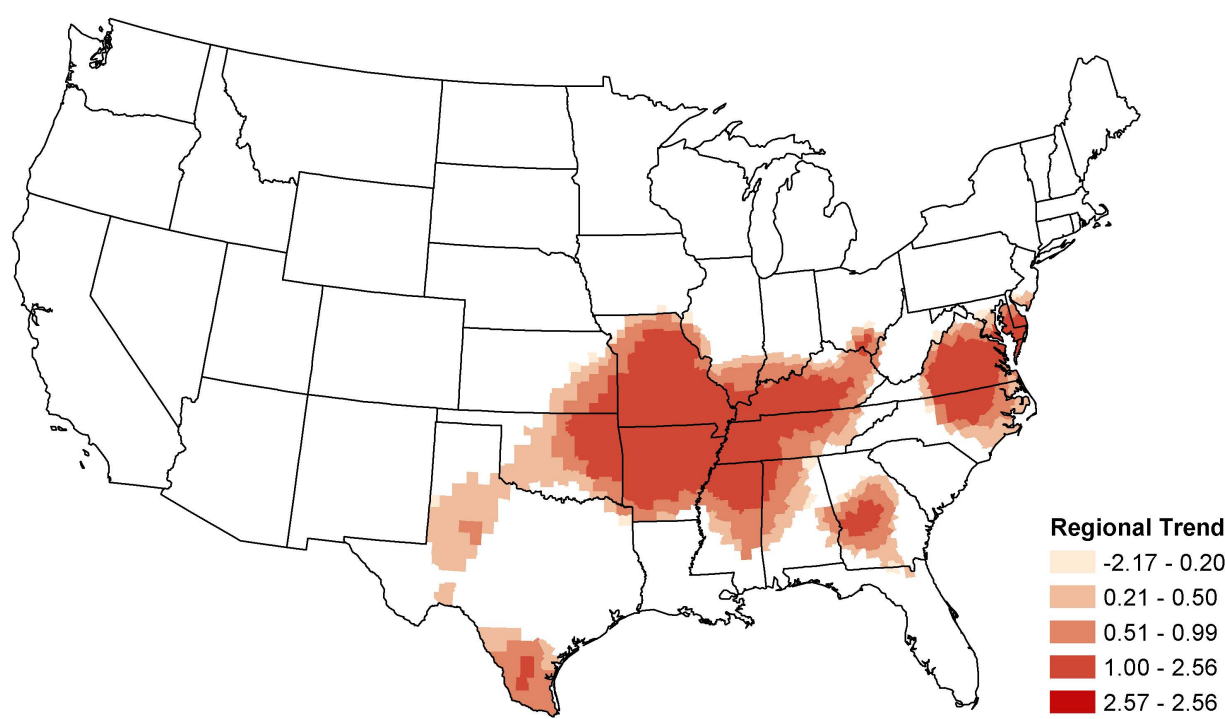

a

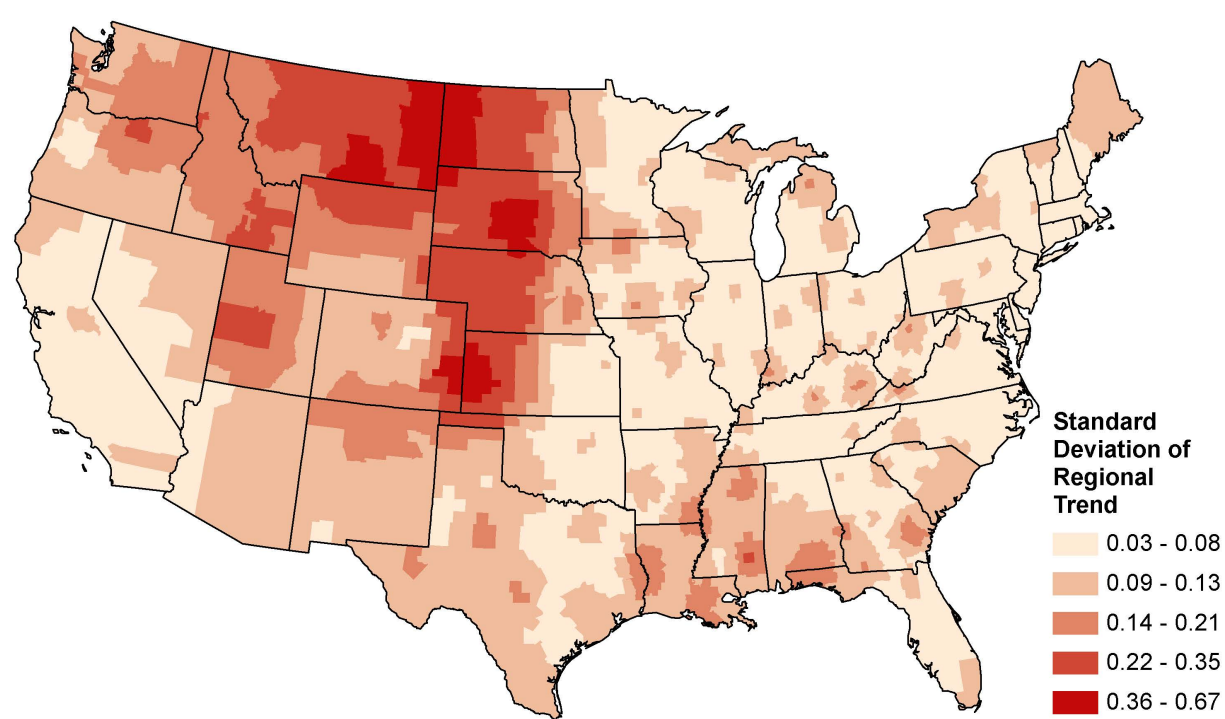

b

Figure S2

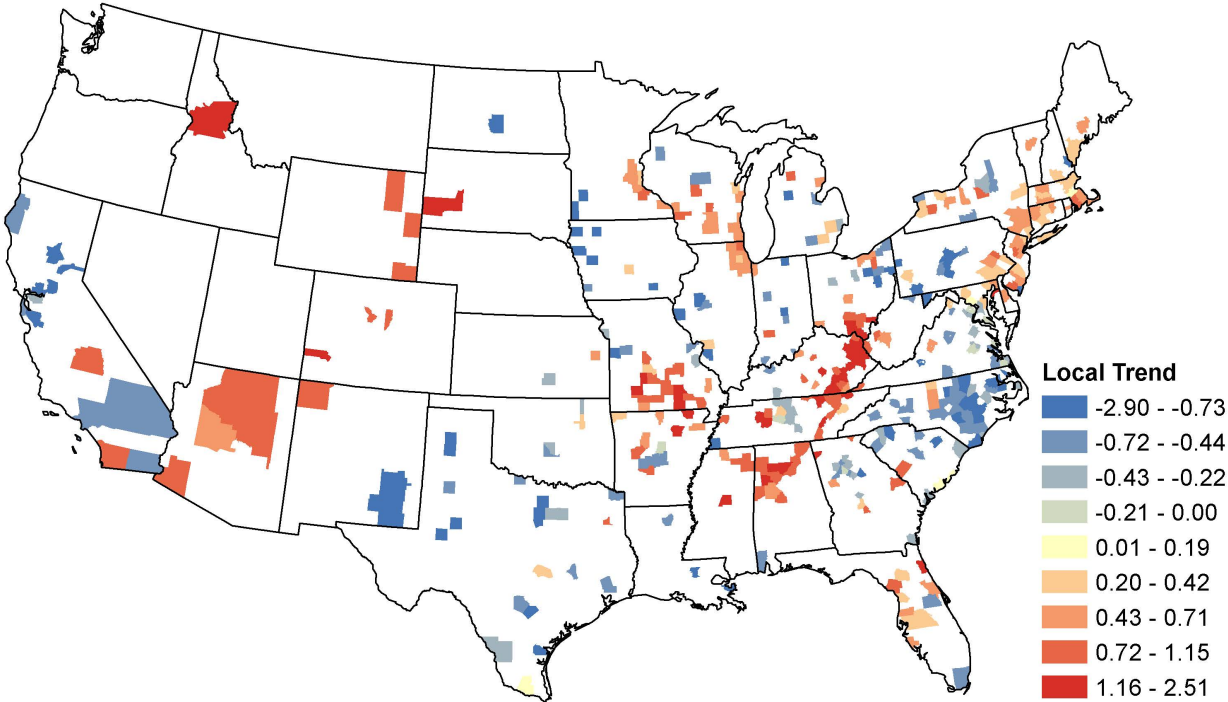

a

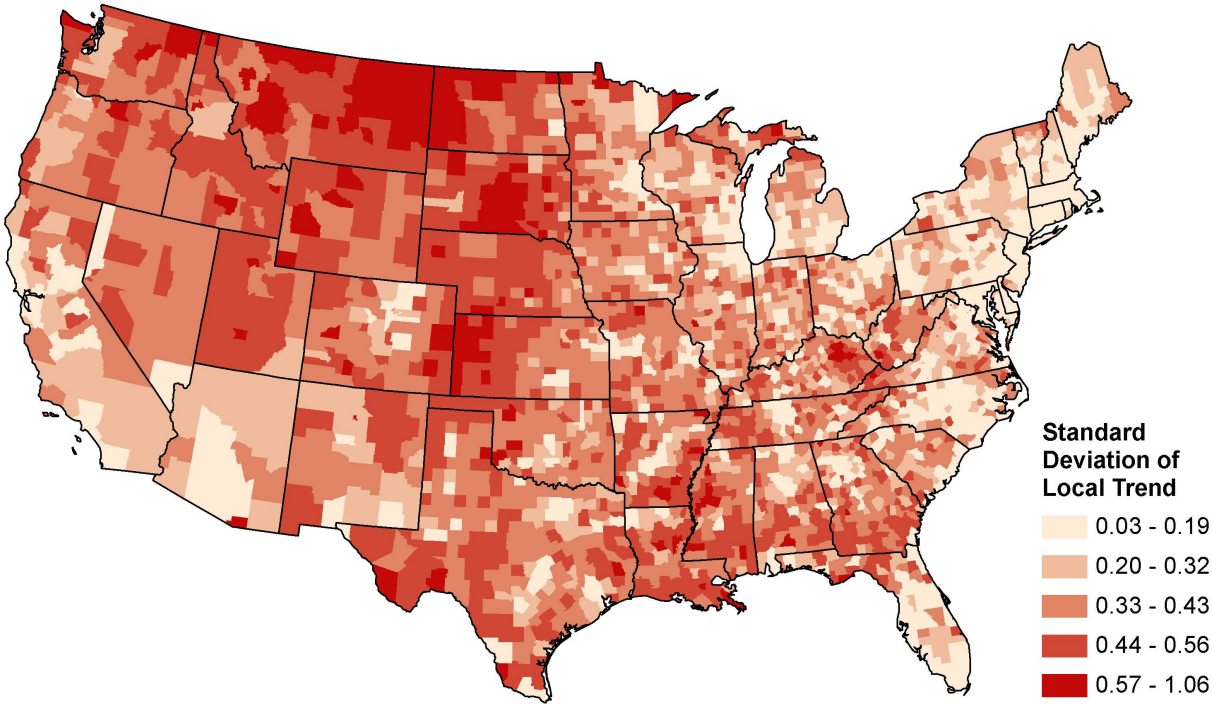

b

Supplement: Supplementary file 1 — Additional file 1: Figure S1. a Posterior mean values of the regional temporal trend parameter for canine Ehrlichia spp. seroprevalence for counties in which the 99% credible interval was strictly positive. b The posterior standard deviation of the regional temporal trend parameter, $\beta_{s}$βs from Equation 2 for all counties. Figure S2. a Posterior mean values of the local temporal trend parameter $\alpha_{1s}^{{}}$α1s for canine Ehrlichia spp. seroprevalence for counties in which the 99% credible interval did not contain zero. b Posterior standard deviation of the local temporal trend parameter for all counties. [file 13071_2020_4022_MOESM1_ESM.pdf]
